# Functional Heterologous Expression of Mature Lipase LipA from *Pseudomonas aeruginosa* PSA01 in *Escherichia coli* SHuffle and BL21 (DE3): Effect of the Expression Host on Thermal Stability and Solvent Tolerance of the Enzyme Produced

**DOI:** 10.3390/ijms21113925

**Published:** 2020-05-30

**Authors:** Ingrid Yamile Pulido, Erlide Prieto, Gilles Paul Pieffet, Lina Méndez, Carlos A. Jiménez-Junca

**Affiliations:** 1 Biosciences Doctoral Program, Universidad de La Sabana, km 7 Autopista Norte, Chía 250001, Colombia; ingridpuma@unisabana.edu.co; 2 Agro-industrial Processes Research Group, Engineering Faculty, Universidad de La Sabana, km 7 Autopista Norte, Chía, Cundinamarca 250001, Colombia; erlide.prieto@unisabana.edu.co (E.P.); linamenca@unisabana.edu.co (L.M.); 3 Science Faculty, Universidad Antonio Nariño, Calle 58 A # 37–94 Bogotá D.C.111511, Colombia; gp.pieffet@uan.edu.co; 4 Bioprospecting Research Group, Engineering Faculty, Universidad de La Sabana, km 7 Autopista Norte, Chía, Cundinamarca 250001, Colombia

**Keywords:** *Pseudomonas aeruginosa* lipase, lipase LipA, overexpression of *E. coli* SHuffle, lipase and foldase overexpression, expression of disulfide bond proteins

## Abstract

This study aimed to express heterologously the lipase LipA from *Pseudomonas aeruginosa* PSA01 obtained from palm fruit residues. In previous approaches, LipA was expressed in *Escherichia coli* fused with its signal peptide and without its disulfide bond, displaying low activity. We cloned the mature LipA with its truncated chaperone Lif in a dual plasmid and overexpressed the enzyme in two *E. coli* strains: the traditional BL21 (DE3) and the SHuffle^®^ strain, engineered to produce stable cytoplasmic disulfide bonds. We evaluated the effect of the disulfide bond on LipA stability using molecular dynamics. We expressed LipA successfully under isopropyl β-d-1-thio-galactopyranoside (IPTG) and slow autoinducing conditions. The SHuffle LipA showed higher residual activity at 45 °C and a greater hyperactivation after incubation with ethanol than the enzyme produced by *E. coli* BL21 (DE3). Conversely, the latter was slightly more stable in methanol 50% and 60% (t½: 49.5 min and 9 min) than the SHuffle LipA (t½: 31.5 min and 7.4 min). The molecular dynamics simulations showed that removing the disulfide bond caused some regions of LipA to become less flexible and some others to become more flexible, significantly affecting the closing lid and partially exposing the active site at all times.

## 1. Introduction

Lipases are heterogeneous enzymes with a high demand in biotechnological processes [1,2,3]. They represent the fourth most important group of enzymes in the global market after carbohydrases, proteases, and phytases [4].

The lipase A (LipA) from *P. aeruginosa* is an enantioselective and organic solvent tolerant enzyme with a high potential use in non-aqueous chemical reactions [5,6]. LipA has a disulfide bond that, while not essential for its activity, is necessary for its secretion through the outer membrane [7,8].

Since *P. aeruginosa* is an opportunistic pathogenic bacteria with a tightly controlled expression of the lipA gene, potential biotechnological applications often require the production in safer hosts [9]. Heterologous expression using *E. coli* has been the preferred strategy for the production of this enzyme due to its easy growth and low production costs [10,11,12]. Nevertheless, the expression in this microorganism often results in aggregated protein production besides the active enzyme [13,14].

LipA belongs to the family I.1 and thus requires a foldase Lif (Lipase-specific foldase) to form an active fold. Both genes *lipA* and *lif* are coupled translationally in the same operon, and a 1:1 interaction between these proteins is necessary to get the correct folding of the enzyme [15,16]. Different approaches have been used for the heterologous expression of *lip* and *lif.* Both genes are expressed in separate plasmids and hosts, and the solubilized enzyme is incubated with the foldase. Another possibility is to insert each gene in a different plasmid but inside the same bacterium [11,12,17,18]. Alternatively, both genes are cloned into the same vector under the control of one or two promoters for the in vivo expression of the functional enzyme [10,17].

However, in all attempts mentioned above, the entire sequence of LipA was cloned and expressed in *E. coli* BL21 (DE3), resulting in a protein fused with its signal peptide which appears not to be recognized by the *E. coli* peptidases. Thus, the enzyme is recovered into the bacterial cytoplasm [10,11,19]. LipA is produced as a soluble protein but, at the same time, non-soluble aggregates are also formed, as previously reported [15,17]. Additionally, the yield is not as high as in native bacteria, probably due to the additional and highly hydrophobic signal peptide fused to the mature protein, which could contribute to non-optimal folding and lower activities [11,12]. Ogino et al. produced LipA in *E. coli* with and without its signal peptide as inclusion bodies that were then solubilized and refolded with the aid of the truncated foldase [12]. They demonstrated that the mature lipase, once refolded, almost doubled the activity of the LipA fused with its signal peptide [12].

LipA in *E. coli* BL21 (DE3) is produced within the cytoplasm and folded without the disulfide bridge. In *E. coli,* this bond is formed stably in the oxidative environment of the periplasm through the disulfide bond formation (DSB) protein system. The use of this system requires the fusion with signal peptides, driving the protein through the Sec, Srp, and Tat systems up to the periplasm [20]. Currently, the use of strains of *E. coli* with mutations has focused on forming and maintaining disulfide bonds inside the cytoplasm. For example, *E. coli* SHuffle^®^ is an engineered strain with knockout mutations in the proteins responsible for the transfer of the reducing potential from NADPH+ to the thiol-disulfide oxidoreductases routes [21]. In this way, thioredoxins and glutaredoxins cannot return to their reduced state. Thus, thioredoxin 1 in its oxidative state in proteins with a pair or more of cysteines can now mediate the formation of thiol groups between two cysteines to form a disulfide bridge [21,22]. Another mutation in the AhpC protein confers the ability to reduce glutareductase 1, maintaining the reducing capacity of the cytoplasm. Moreover, SHuffle expresses the chaperone disulfide bond isomerase (DsbC) inside the cytoplasm to reshape disulfide bonds in proteins with two or more disulfide bridges [21].

The significance of the disulfide bond on the stability of proteins in the presence of environmental stressors such as elevated temperatures and solvents has been demonstrated [23,24,25]. Likewise, the inclusion of disulfide bonds resulted in more thermostable proteins than their wild type counterparts in different approaches for upgrading proteins [26,27,28]. In a recent investigation, the insertion of a disulfide bond through site-directed mutagenesis approach into the thermostable enzyme of *Geobacillus thermocatenulatus* did not affect either its enzymatic activity or its thermotolerance relative to the wild enzyme. The engineered disulfide bond formed with the inclusion of two cysteines, one in the lid and the other in the core, became a physical restrain that causes the enzyme to be in a permanent open form and thus in the active conformation. Nevertheless, the changes between the mutant and the native enzymes were evidenced when they were pretreated with the detergents Triton X-100 and hexadecyltrimethylammonium bromide (CTAB), showing that the mutant exhibits more hyperactivity and resistance to oxidants such as copper chloride than the native enzyme [29]. 

The effect of the disulfide bond on the stability of the lipase LipA in hydrophilic solvents and high temperatures has not been addressed yet. The production of recombinant enzymes, especially those under the control of strong promoters like T7, disturbs the delicate balance between the soluble and aggregated forms in an already overloaded bacterial cytoplasm, driving the expressed proteins to aggregation [14]. However, some approaches can reduce the precipitation of recombinant proteins and favors their soluble production. Such strategies comprise the growth of cultures at low temperatures, low inductor concentrations, low copy number plasmids for gene expression, fusion partners, and the coexpression with chaperones [30,31]. Currently, a strategy to produce recombinant proteins regulated by T7*lac* promoters is through the autoinducing medium [32]. The rationale of this method is related to the differential use of carbon sources in the medium, such as glucose, glycerol, and lactose, allowing the expression of plasmid-borne genes once the glucose has depleted. At the same time, other ingredients in the culture, including buffer salts, magnesium, and trace metals, help to maintain the growth conditions to reach a considerable cellular density [32]. The autoinducing culture has shown a better production of recombinant proteins derived from higher bacterial densities and a more controlled expression of the recombinant genes regulated by the T7 promoter than those reached with IPTG induction [32]. Additionally, a successful production of Sec-system-dependent membrane proteins for periplasmic secretion was made with Lysogenic broth without IPTG induction [33]. Similarly, the leaking effect permitted the successful expression of recombinant genes, probably due to lower rates of protein production than those achieved with IPTG [33]. Despite the approaches mentioned above, some enzymes such as LipA, that are prone to be precipitated in *E. coli* require other strategies focused on the coding gene which allow achieving the correct folding and, therefore, improving the activity from this host.

This study aimed to express a heterologous mature lipase LipA from *P. aeruginosa* in a soluble, stable, and active form. To achieve this goal, we cloned the sequences corresponding to mature LipA and a fragment of its cognate Lif, the latter without the sequence for anchoring in the inner cell membrane. We used a dual vector with a low copy number, and each gene was under the control of the T7*lac* promoter. Furthermore, we used two *E. coli* expression strains: *E. coli* SHuffle, a microorganism engineered to form cytoplasmic disulfide bridges, and the conventional *E. coli* BL21 (DE3) K12 strain unable to form disulfide bonds stably inside the cytoplasm. Both strains exhibit some variations in their genetic background related to the expression of recombinant genes. They were grown at low temperatures and with low stirring; the activity of the lipases produced in both strains was compared after induction with IPTG at two optical densities and with an autoinducing medium [32]. We evaluated the enzymes produced by each strain, their resistance to different temperatures, and their tolerance to methanol and ethanol. We found changes in the activity and tolerance to temperature and solvents relating to the host used.

To connect the simulations more closely to the experiments and establish the effect of the disulfide bridge on the structure of our enzyme, we made long molecular dynamics (MD) simulations with two models of LipA, one with and the other without the disulfide bridge. The simulations showed, in the case of LipA with the disulfide bridge, a stable core with parts of the lid structure highly flexible, especially the helices alpha 5, G2, and their connecting loops. Removing this covalent bond increased the overall flexibility of the lipase in specific regions that were already flexible while making other parts more rigid. LipA without the disulfide bridge revealed alterations of the opening and closing mechanisms of the enzyme and other structures near to the LipA entrance, which could influence the stability of the protein against solvents as well as its temperature tolerance. 

## 2. Results 

### 2.1. Cloning of LipA and its Foldase

**Figure 1:**
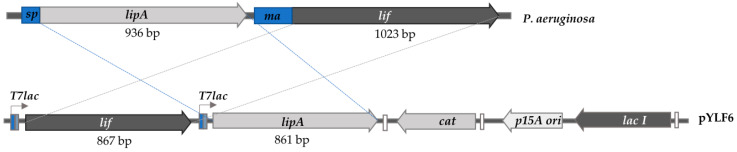
Construct pYLF6 (pACYC-Duet-1*-lipA-lif*) with sequences of the mature *lipA* (861 bp) without signal peptide (*sp*) and *lif* (867 bp) without an inner membrane anchor (*ma*) inserted in the pACYC-Duet-1 (Novagen). Both genes are preceded for the T7*lac* promoter. *cat***:** chloramphenicol resistance gene; p15 ori: the origin of replication of p15; *lacI*: gene codifying LacI

The nucleotide sequences from the *lipA* and *lif* genes of *P. aeruginosa* PSA01 were similar to those already stored in public databases. The *lipA* sequence with 936 nucleotides displayed 99% of identity with the lipase *lipA* from *P. aeruginosa* PAO581 (AGV61140.1) and *lip 9* from *P. aeruginosa* LST03 (BAF92628.1) [34]. There were three different nucleotides with this last sequence, one of them coding for a distinct amino acid (I130V). The *lif* sequence of PSA01 with a length of 1023 bp exhibited 100% identity with *lif* 9 of *P. aeruginosa* LST03 (BAF92629.1). It was 99% identical to the *lif* sequence from *P. aeruginosa* ATCC 27853 (ANT75757.1). The mature sequence of LipA, inserted in the multi cloning site two (MCS2) without the native signal peptide and an additional codon for methionine, had 861 bp, which encoded a protein with 285 residues (Figure 1). This protein had a molecular weight of 30.1 KDa. The sequence of the gene coding Lif without 156 bp at the N terminal end and with a length of 867 bp was inserted in the MCS 1 (Figure 1). The *T7lac* promoter controlled each gene. The molecular weight for Lif, calculated without the initial methionine, corresponded to 32.4 KDa (Snapgene, GSL Biotech, Chicago, IL, USA). The recombinant plasmid pACYC-Duet-1 with both genes was named pYLF6.

#### Nucleotide Accession Number

The nucleotide accession number for the lipase gene *lipA* reported here is Genbank: MK336958; for the foldase gene *lif* is GenBank: MK336959.

### 2.2. Monitoring of the Recombinant LipA Expression in E. coli SHuffle and E. coli BL21 (DE3) Strains.

#### 2.2.1. Induction with IPTG

**Figure 2:**
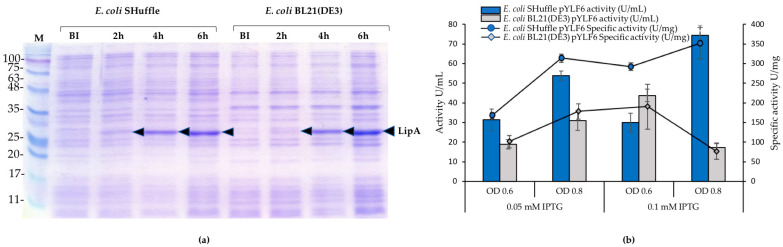
Time course of induction with IPTG and the activity of LipA produced by recombinants of *E. coli* with the plasmid pYLF6. (**a**) SDS-PAGE of samples of *E. coli* SHuffle and *E. coli* BL21 (DE3) with pYLF6 induced with 0.05 mM of IPTG; the samples were taken before induction (BI) and at the intervals indicated. (**b**) Activity (U/mL) and specific activity (U/mg) of LipA in each strain after 6 h of induction at two IPTG concentrations and two optical densities (OD_600_). M: Opti-Protein XL protein weight marker G266 (ABM Inc, Richmond, BC, Canada).

We incubated both *E. coli* strains with the construct pYLF6 at a temperature of 18 °C and 100 rpm to achieve a slow growth. Figure 2a shows the SDS-PAGE profiles of whole culture aliquots (supernatant and cells) after induction with 0.05 mM and 0.1 mM of IPTG. It shows the overexpression of LipA after 2 h of induction, and it increases in the following 4 and 6 h. The overexpression of just one protein near to 30 KDa allows us to assume that it corresponds to LipA. Figure 2b presents the activity of the enzyme produced by each strain after 6 h post-induction. In the case of the LipA from *E. coli* SHuffle, there is a notable variation in the activities measured at an optical density at 600 nm (OD_600_) of 0.8 with 53.9 and up to 74.4 U/mL upon induction with 0.05 mM and 0.1 mM of IPTG, respectively. The activities obtained with induction at OD_600_ of 0.6 did not show changes under the two IPTG concentrations used.

In contrast, the activities of LipA produced by *E. coli* BL21 (DE3) are lower than those observed with the SHuffle strain. The maximum activity of 43.8 ± 3.2 for *E. coli* BL21 (DE3) was obtained with an IPTG of 0.1 mM and at an absorbance of 0.6. The production of soluble proteins was similar for both organisms, but the specific activity was higher in the *E. coli* SHuffle than in the *E. coli* BL21 (DE3) (Figure 2b). Protein aggregates were observed in all cultures from both strains.

#### 2.2.2. Induction with Autoinducing Medium

**Figure 3:**
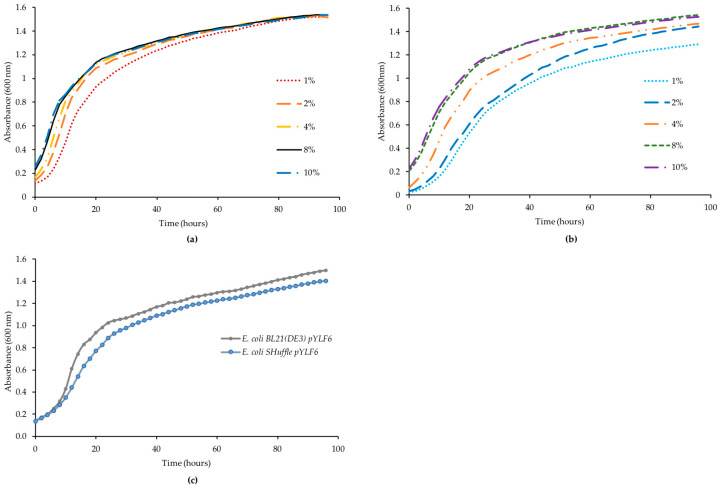
Growth curves for strains using the autoinducing medium at 18 °C slow agitation and five different inocula. (**a**) *E. coli* BL21 (DE3) with pAYLF6. (**b**) *E. coli* SHuffle with pAYLF6. (**c**) Growth curves of both strains in the autoinducing medium inoculated at 4%. Inocula were grown ON at 30 °C and 100 rpm in the non-autoinducing medium.

Figure 3 presents the effect of the auto-inducing medium on the growth of strains. The growth curves reveal minor differences among inocula, although some differences and effects were observed between the *E. coli* SHuffle (Figure 3b) and the *E. coli* BL21 (DE3) (Figure 3a). *E. coli* BL21 (DE3) with pYLF6 had a slightly faster growth (µ= 0.12 h^−1^; doubling time= 6.7 h) than *E. coli* SHuffle pYLF6 (µ = 0.13 h^−1^; doubling time= 7.2 h) (Figure 3c). Although we used the autoinducing medium, the densities did not reach absorbances higher than 1.6, as was expected with this medium.

**Figure 4:**
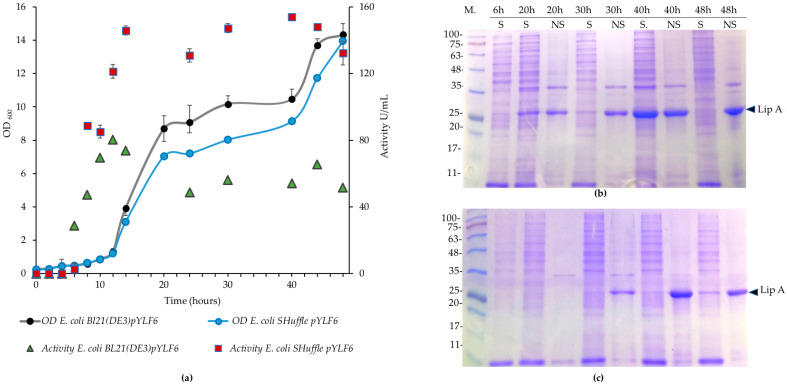
Growth and enzymatic activity of the recombinants *E. coli* BL21 (DE3)-pYLF6 and *E. coli* SHuffle-pYLF6 in the autoinducing medium. (**a**) Growth curves of *E. coli* strains and activities of the enzymes at specific times in autoinducing medium at 18 °C and 100 rpm for 48 h. (**b**) Protein profiles in SDS-PAGE at specific times during growth cultures from *E. coli* BL21 (DE3) pYLF6 of soluble (S) and insoluble (NS) fractions. (**c**) SDS-PAGE protein profiles of *E. coli* SHuffle at the same conditions. M: Opti-Protein XL protein weight marker G266 (ABM Inc, Richmond, BC, Canada).

Figure 4 shows the production and activity of lipase using the autoinducing broth. The lipase activity was registered from 6 h, being 28.5 ± 3.4 U/mL for *E. coli* BL21 (DE3) and 2.65 ± 1.3 U/mL for *E. coli* SHuffle (Figure 4a). The activity for *E. coli* BL21 (DE3) was at its maximum (80.3 ± 4.1 U/mL) at 12 h and decreased progressively until the end of the second day. In contrast, the activity for SHuffle increased from 8 h up to the end of the experiment, with a maximum activity of 154 ± 3.0 U/mL at 40 h. The amount of insoluble fractions increased with time in both cultures, as evidenced by the SDS-PAGE (Figure 4b,c). Comparisons of the soluble and insoluble fractions show that lipase is the main protein present in the insoluble fractions obtained in each culture. Despite the activities of the LipA produced by the strain SHuffle, a band with the size expected for the lipase was observed just until 30 h (Figure 4c).

The expression level of lipase using the recipe ZYM 5052 without lactose was evaluated in each host (Table 1). Both strains were able to produce LipA, showing that they had an uninduced expression of the enzyme from the early phases of the growth. However, the activities of *E. coli* BL21 (DE3) were greater than those exhibited by *E. coli* SHuffle in the first 13 h. Subsequently, the latter passed the activities of *E. coli* BL21 (DE3) after 18 h (Table 1).

### 2.3. Stability in Methanol and Ethanol of LipA Produced by E. coli SHuffle and E. coli BL21 (DE3)

**Figure 5:**
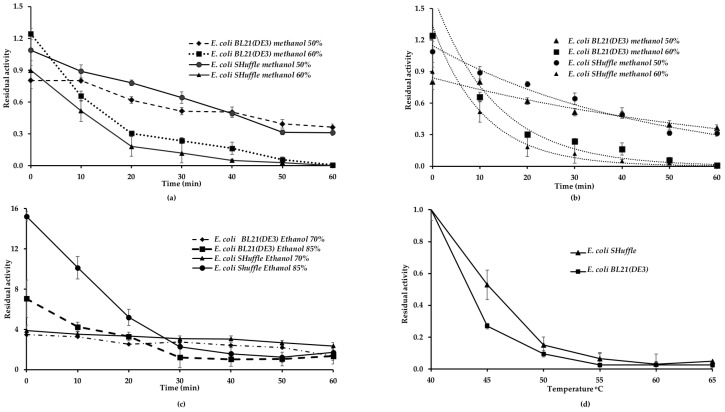
Stability in methanol and ethanol and temperature of LipA produced by *E. coli* BL21 (DE3) pYLF6 and *E. coli* SHuffle pYLF6. (**a**) Residual activities in methanol 50% and methanol 60%. (**b**) Deactivation curves in methanol 50% and methanol 60%, adjusted to the exponential decay model. (**c**) Residual activities in ethanol 70% and 85%. (**d**) Residual activities of LipA at five temperatures (considering the activity at 40 °C as reference). All experiments were carried out for one hour.

Figure 5 shows the stability of LipA produced for each strain towards two concentrations of methanol and ethanol. After one hour of exposure to methanol, the residual activity of the enzymes fell rapidly, though the instability was more significant for methanol 60% (Figure 5a). The deactivation profiles followed an exponential model from which it was possible to estimate the half-life for this enzyme in methanol (Figure 5b). The t½ was 31.5 min and 7.4 min for methanol 50% and 60%, respectively, for *E. coli* SHuffle LipA. The t½ was 49.5 min and 9 min in the same solvent and concentrations for LipA produced by *E. coli* BL21 (DE3).

LipA was very stable in ethanol, but some differences could be appreciated depending on the host that produced it. The behavior of both enzymes was quite similar to ethanol 70%, and the enzymes exhibited twice as much activity as the enzyme without ethanol (Figure 5c). When the enzymes dissolved in ethanol at a concentration of 85%, both enzymes exhibited hyperactivation. However, the lipase from *E. coli* SHuffle showed a higher hyperactivation during the first 20 min of incubation. The hyperactivation decreased rapidly, but both enzymes conserved more than twice the activity during the period evaluated.

### 2.4. Stability of LipA Produced by E. coli SHuffle and E. coli BL21 (DE3) to Temperature 

The thermal stability of the enzymes produced by each *E. coli* strain was evaluated through six different temperatures for one hour. LipA is naturally produced by *Pseudomonas aeruginosa*, a mesophilic organism, so it is stable at temperatures below 40 °C. Both enzymes were unstable, and the residual activity decreased as the temperature increased (Figure 5d). The residual activity of the LipA from the *E. coli* SHuffle was higher than 50%, while the *E. coli* BL21 (DE3) LipA exhibited just 27% at 45 °C compared with the activity at 40 °C. Both enzymes retained only 20% of their initial activity at 50 °C, and they lost their activities at 60 °C.

### 2.5. Flexibility of LipA with and without Disulfide Bonds during the Molecular Dynamics Simulations 

**Figure 6:**
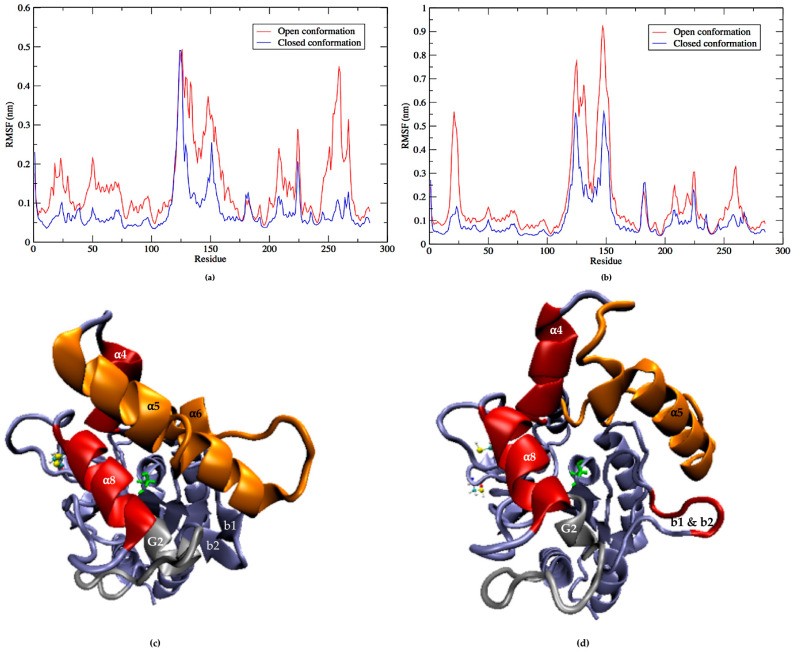
Root Mean Square Fluctuation (RMSF) and performance of lid secondary structures of LipA. (**a**) RMSF during the molecular dynamics (MD) simulations of LipA with the disulfide bridge in the open and closed conformations. (**b**) RMSF values for LipA without the disulfide bridge in the open and closed conformations. (**c**) 3D secondary structures of the LipA lid with a disulfide bridge during the MD simulations; the catalytic serine (green) and the cavity are protected by the coordinated and oriented approach of the lid structures. (**d**) 3D secondary structure of LipA without the disulfide bond showing the increased flexibility and altered orientation of the helix α5 -loop-helix α6. The small ß-strands b1 and b2 increased their flexibility with respect to the LipA with the disulfide bridge. A partially exposed cavity is observed during the MD simulation.

Molecular dynamics simulations of LipA with the disulfide bridge showed that the two most flexible parts were the helices α5 (residues 125 to 147), α6 (residues 155 to 163), and G2 with connecting loops (residues 251 to 268); together, they are responsible for the opening and closing of the active site (Figure 6a). The helix α5 is considered part of the lid along with α4 and α6 [35], sometimes with the helix α8 (residues 210 to 219) as a second lid [36]. While it is true that the helix α8 shows some level of flexibility during the simulation, it is the helix G2 and its connecting loops that move towards the helix α5. They are therefore responsible for closing the catalytic cleft, and together with the helix α5, which also moves towards them, they form the actual lid domain that protects the active site in a coordinated movement with the correct orientation (Figure 6c). 

**Figure 7:**
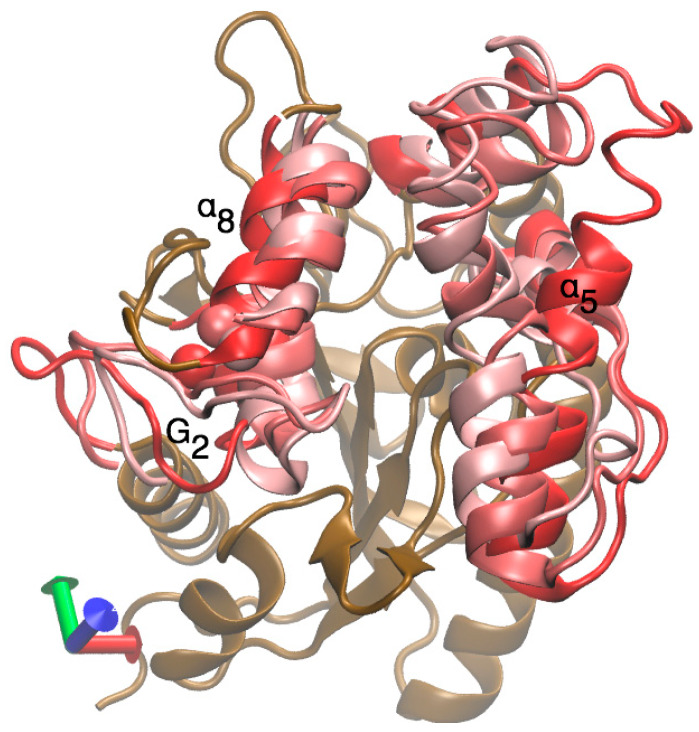
Superimposed structure of the helix α5 G2, and α8 of LipA with the disulfide bridge, as produced by *E. coli* SHuffle at 0 ns (red), 30 ns (dark pink), and 60 ns (light pink).

The performance exhibited by the LipA without the disulfide bond, which corresponds to the protein produced in the cytoplasm by *E. coli* BL12 (DE3), was very different. Removing the disulfide bridge increased notably the flexibility of the helix α5 -loop-helix α6 (residues 125 to 154 and 155 to 163 in Figure 6b and helices in orange in Figure 6d), affecting both their internal stability and their orientation with respect to the active site. Hence, removing the disulfide bond increased the flexibility of LipA in specific secondary elements that were already very flexible, such as the loop-α5-loop-α6 and the b1-b2 elements, which in turn affected their coordinated movement and consequently the closing mechanism. Surprisingly, other secondary structure elements became less flexible without the disulfide bond. Such is the case with the G2 helix and its connected loop, which forms an essential part of the closing mechanism of the LipA form with the disulfide bond (colored in gray in Figure 6c). This structure showed a much smaller flexibility and contributed to the uncovering of the active site cavity during most of the simulation. The movement of the helix α5, G2, and α8 is shown in Figure 7, in which their conformations are represented at different moments in times using a color code: t = 0 nanoseconds (ns) (red), 30 ns (dark pink) and 60 ns (light pink). 

## 3. Discussion

LipA from *P. aeruginosa* is a lipase with recognized characteristics, such as its enantioselectivity, solvent resistance, and tolerance to temperatures up to 40 °C. It is useful in hydrolysis and synthesis reactions for a wide range of substrates [9,17,37,38]. However, lipase production by the native microorganism depends on various physiological and nutritional factors, some of which have not yet been elucidated [7,39]. Due to the opportunistic pathogen nature of *P. aeruginosa*, the LipA expression has been done mainly through heterologous expression from harmless organisms like *E. coli* [10,11,17]. The active heterologous expression of this enzyme requires the concomitant expression of its foldase, making it even harder to explore new applications [40]. In this investigation, we cloned *lipA* and *lif* in the plasmid pACYC-Duet-1, a dual plasmid used for the coexpression of two genes, each controlled by the T7*lac* promoter. Contrasting with previous approaches, we removed the highly hydrophobic leader peptide of LipA, a sequence non-recognized by *E. coli;* in this way, we are contributing to the expression of a protein less prone to aggregate. Further, we removed 58 amino acids from the N terminus of the foldase gene, a highly hydrophobic sequence not related to its activity but involved in its union with the inner membrane of the native bacteria [12,40]. With this construct, we could produce a functional LipA, as was evidenced in the SDS-PAGE gels and the activity obtained with p-nitrophenyl palmitate.

**Figure A1:**
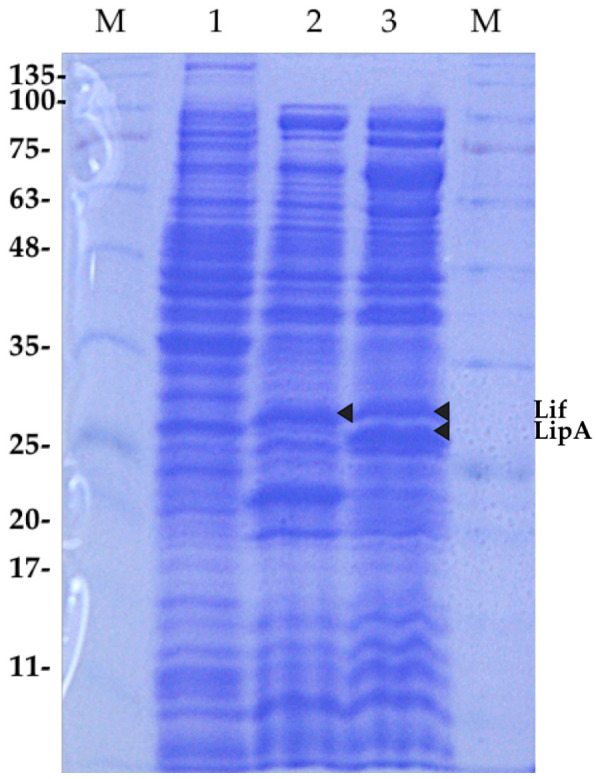
SDS PAGE protein profile of crude lysates of *E. coli* BL21 (DE3) after induction for 24 h with IPTG 0.1 mM. Twenty nanograms of proteins were loaded in each lane. 1: *E. coli* BL21 (DE3); 2: *E. coli* BL21 (DE3) with pACYC-*lif*; 3: BL21 (DE3) with pYLF6. M: Opti-Protein XL protein weight marker G266 (ABM Inc., Richmond, BC, Canada).

Contrary to Ogino and others, who reported deficient but detectable amounts of foldase, we did not observe foldase in the SDS-PAGE gels at the conditions evaluated [10,11,17]. Assuming that Lif underwent the removal of the initial methionine, this protein would have 288 residues corresponding to a molecular weight of 32.4 KDa (Snapgene, GSL Biotech), precisely the size observed when it expresses in *P. aeruginosa*. Even though a strong promoter preceded this gene as T7*lac*, we could not detect it in the soluble nor the insoluble fractions. We suppose that *lif* suffered some regulation during its transcription or translation in the *E. coli* strains we used, which significantly disrupted the appropriate production of the protein and, in turn, affected the folding of LipA. We cloned just the *lif* gene in the pACYC-Duet-1 system (pACYC-*lif*), and it was transformed in *E. coli* BL21 (DE3). The SDS-PAGE profile of the crude extracts of this strain was compared with the protein profile of *E. coli* BL21 (DE3) pYLF6 after 24 h of inductions with IPTG (Figure A1, Appendix A). We observed a band which may correspond with the overexpressed foldase. These findings allow us to infer that Lif is produced only in small quantities sufficient for the folding of a certain number of lipase molecules, resulting in the aggregation of the remaining enzyme. Other reports with foldases from *P. cepacia* or *Ralstonia* sp. have revealed similar findings, with the non-observable or low production of the chaperone despite the strong promoter upstream of the gene and the replacement of part of the high guanine-cytosine N terminal sequence by another one with an optimized codon sequence for expression in *E. coli* [19,41].

We used culture conditions for a slow expression of LipA that would favor its correct folding and solubility [30]. Accordingly, we used a temperature of 18 °C for the incubation of the cultures, 100 rpm of orbital agitation, and a plasmid with a low copy number, as well as two methods of induction to control the growth rate. However, we observed the production of insoluble fractions that corresponded to the recombinant lipase in all experiments. It is possible to use other strategies to increase the amount of soluble lipase produced in this host such as the codon optimization for both *lipA* and *lif,* which are highly biased in the use of guanine and cytosine ((LipA 66.3% and Lif 67.9%; www.genscript.com/tools/rare-codon-analysis) with respect to the one used by *E. coli* [42].

When the cells were induced with IPTG, we observed that the insoluble fractions of the protein were higher than those found when we used the autoinducing medium, as reported for other proteins [32,43]. This fact could be attributed to a lower expression of genes controlled by T7*lac*, which might result in the lower production of recombinant proteins, thus avoiding the overcrowding of the protein in the cytoplasm and facilitating its correct folding. We believe that this medium was helpful for our system regulated by the *lac* operon, with two genes expressed at the same time, which may need special conditions for both induction and harvesting [32].

We produced culture broths using flasks, high cell densities, and higher amounts of lipase comparable with those produced by Studier with the autoinducing medium [32]. However, the growth of each strain exhibited differences, probably due to the genetic backgrounds displayed. For instance, the genetic modification in SHuffle, which affects the cytoplasm oxidation state, could result in a slower growth rate than that observed in BL21 (DE3), which eventually, besides the formation of the disulfide bridge, resulted in a more controlled and higher activity (Figure 4) [22].

Furthermore, both strains have variations related to the regulation of the *lac* operon system that could affect their growth in this medium. The promoter that controls the expression of the chromosomal T7 RNA polymerase in *E. coli* BL21 (DE3) is the leaky promoter LacUV5, which drives to the T7 RNA polymerase transcription in the early stages of the culture. As we observed with this *E. coli* strain, the active lipase production reached a peak at 28 h but fell afterwards, despite a high production of protein in the last stages of the culture. It is possible that the high expression of the protein in a shorter timeframe affected the appropriate folding, leading to precipitation as inclusion bodies, as we observed in insoluble fractions in the last stages of the culture for this strain (Figure 4). On the contrary, the activity in *E. coli* SHuffle, with the T7 RNA polymerase under the *lac* promoter control and additional doses of LacI in its chromosome (*lacI*^q^), exhibited lower values at first stages of the culture but increased until the end of the culture. This result suggests a more delayed and controlled expression with SHuffle, allowing the appropriate folding and activity of the lipase produced by this strain. 

The differences in both strains regarding the leak or uninduced expression were observed easily by growing both strains in the autoinduction medium without lactose. The activity obtained for *E. coli* BL21 (DE3) was higher during the first hours of culture than that measured in SHuffle, but soon after the activity of *E. coli* SHuffle increased steadily and surpassed the activities from BL21 (DE3) (Table 1). The presence of low amounts of lactose in the medium, which is a contaminant often found in the nitrogen source tryptone, could result in a more controlled expression of genes in both strains [44]. To summarize, we observed a more regulated expression of the promoters under the control of lactose in *E. coli* SHuffle, which permitted the expression and folding of a higher amount of the enzyme.

*E. coli* SHuffle is an ideal recipient for expressing *lipA* in the cytoplasm, considering the presence of a disulfide bond in this protein. This covalent bond has an important role in the thermal stability of lipase and, its resistance to proteases, and it also confers a high activity and stability in ethanol compared to the enzyme without this bond [7]. We found that LipA is stable in high concentrations and suffers a hyperactivation in ethanol, which was accentuated for the enzyme with the disulfide bond. The stability of lipases of *P. aeruginosa* and their hyperactivation with ethanol has been recognized before [17,37]. This is attributed to the high rigidity of the structure, which also confers tolerance to high temperatures. However, LipA from *E. coli* BL21 (DE3) showed a somewhat better activity in methanol than SHuffle. Our findings highlight the importance of the disulfide bond not only in the activity of the enzyme but also in its stability in hydrophilic solvents such as methanol and ethanol. 

Although the *E. coli* strains used in this study have subtle genetic differences as stated above, mutations affecting the cytoplasmic redox potential and the maintenance of disulfide bonds could play an essential role in the differences we found between the enzymes produced [21,22]. In BL21 (DE3) cells, the maintaining of the disulfide bonds is tightly inhibited by the glutathione redox system which acts as a thiol buffer [45]. Thus, the proteins requiring this covalent bond must be driven to the periplasm [46]. 

However, recent studies have confirmed the in vitro disulfide formation with oxidant agents and environmental conditions, such as high dilutions and basic pH, that favoring the oxidation of thiol groups by the molecular oxygen [47]. Similarly, experiments performed with the quadruple mutant BTL2 from *G. thermocatenulatus* expressed in *E. coli* BL21 (DE3), lipase modified by the elimination and addition in a different place of a disulfide bond, demonstrated that the enzyme had assembled the disulfide bond without the prior treatment with oxidant agents [29]. According to the authors, the exposure of the enzyme to detergents and air during the purification process favored the proximity of cysteines and, subsequently, the oxidation of thiol groups and formation of the covalent bridge [29].

With these results, it seems that the extracellular disulfide bond formation will depend on the conjugation of certain conditions, such as the proximity and correct orientation of cysteines, the local structure surrounding the cysteines compromised with the disulfide bond, with its geometric or steric constraints; the amino acids surrounding the cysteines; and other cysteines within the enzyme [48,49].

The MD simulations showed that LipA is composed of a very stable core and a highly flexible lid domain. For the LipA with a disulfide bond, flexibility occurs through the movement of alpha-helices and loops in the lid (loop-α5-loop and loop-G2-loop), allowing or preventing access to the active site while retaining its secondary structure and orientation. In contrast, the simulation of LipA without a disulfide bridge showed that the closure occurred in a less coordinated manner and mainly through the loop between α5 and α8, as it became extremely flexible. The removal of the disulfide bond significantly affected the flexibility of the lid by altering both the stability of the helices and their orientation with respect to the active site, causing this cavity to remain relatively accessible at all times. In the enzyme without a disulfide bridge, the high mobility of the structures surrounding the lid could explain its higher instability with the increase in temperature. Simultaneously, greater flexibility could facilitate the formation of temporary hydrogen bonds or other connections, as mentioned in other studies, which could generate a higher tolerance against methanol induced instability than its counterpart produced by SHuffle [50].

## 4. Materials and Methods

### 4.1. Bacterial Strains, Plasmids, and Reagents.

The *P. aeruginosa* PSA01 was isolated from palm fruit residues. The *E. coli* DH5 alpha and the *E. coli* BL21 (DE3) (*fhuA2 [lon] ompT gal (λDE3) [dcm] ∆hsdS λDE3* =λ sBamHIo ∆EcoRI-B int::(lacI::PlacUV5::T7 gene1) i21 ∆nin5) were acquired from Invitrogen Inc. (Carlsbad, CA, USA), and used for the storage and expression of plasmids with the lipase and foldase genes. The *E. coli* SHuffle K12 (F´ lac, pro, lacIq/Δ(ara-leu)7697 araD139 fhuA2 lacZ::T7 gene1 Δ(phoA)PvuII phoR ahpC* galE (or U) galK λatt::pNEB3-r1-cDsbC(SpecR lacIq) ΔtrxB rpsL150 (StrR) Δgor Δ(malF)3) was a kind gift from Doctor Luis Reyes (Engineering Faculty, Universidad de Los Andes, Bogotá, Cundinamarca, Colombia). 

The total DNA and plasmids were isolated with the EZNA DNA isolation kit and Plasmid Mini kit II (Omega Bio-Tek, Inc., Norcross, GA, USA). The plasmid pGem-T Easy was purchased from Promega (Madison, WI, USA); the pACYC-Duet-1 was acquired from Novagen EMD Millipore (Billerica, MA, USA).

All the microbiological reagents for the growth of recombinants (tryptone, sodium chloride, yeast extract, lactose, glucose, glycerol, ammonium sulfate, magnesium sulfate, sodium phosphate, and potassium phosphate) were acquired from PanReac (Barcelona, Spain). The platinum *Taq* DNA polymerase high fidelity, deoxynucleotide triphosphates (dNTPs), agarose, and restriction enzymes were acquired from Thermo Fisher Scientific Inc (Waltham, MA, USA). Reagents for the analysis of proteins were obtained from Bio-Rad (Hercules, CA, USA). Other reagents used in this study were of analytical grade.

### 4.2. Cloning of lipA and lif

*P. aeruginosa* PSA 01 was grown in Lysogenic broth for 16 h at 37 °C and 200 rpm. The whole DNA was used as a template with the primers PafoR and Parev to amplify the entire gene *lipA* (Table 2). The PCR followed an initial denaturing step at 94 °C for 5 min and followed 30 cycles of 94 °C for 30 s, 58 °C for 30 s, and 72 °C for 1.5 min. The final extension was carried out at 72 °C for 5 min. Each reaction was a mixture of 1.8 mM of MgCl_2_, 0.2 mM of dNTPs, 0.4 µM of each primer, 1% of DMSO, 0.02 U/µL of DNA polymerase, and 2 µL of DNA (approximately 2 ng/µL).

The gene *lif* was amplified with Folfor and Perm at the same thermocycling conditions and reaction mixture explained above for *lipA*, except for the annealing temperature, which was 70 °C, and the use of 2.5% of DMSO. Both genes were cloned in a pGEM-T easy vector and transformed in *E. coli* DH5α (Promega, Madison, WI, USA). The plasmids with the complete sequence of both genes were sequenced through Macrogen Inc. (Seoul, South Korea) and compared with those found in the National Center for Biotechnology Information (NCBI )databases (https://www.ncbi.nlm.nih.gov).

A fragment of *lipA* without the native leading peptide (first 78 nucleotides) was amplified by PCR with the primers NdelipF and XholipR (Table 2). Likewise, a fragment of *lif* was amplified with the primers Ncofol and PBR (without the first 156 nucleotides, which translates a domain of 52 residues associated with its anchorage to the inner membrane in *P. aeruginosa*) [12]. The PCR reaction and cycling conditions were those described above for amplifying the whole gene. The *lipA* amplicon and pACYC-Duet-1 were digested with *Nde*I and *Xho*I, ligated into the multicloning sequence two (MCS 2) of the plasmid, and transformed in chemically competent *E. coli* DH5α [51]. The recombinants were selected on LB plates with chloramphenicol (50 µg/mL). 

After this, the plasmid pACYC with *lipA* (named as pYL6) and the Ncofol-PBR amplicon (codifying foldase) were digested with *Nco*I and *Hind*III, ligated into the MCS 1, and transformed in *E. coli* DH5α [51]. The in-frame location of both genes was verified by sequencing. The final construct with *lipA* and *lif* as inserts, had a final size of 5613 bp, was called pYLF6 (Figure 1), and was transformed in the chemically competent cells, *E. coli* BL21 (DE3) and *E. coli* SHuffle K12.

### 4.3. Expression of lipA in Strains of E. coli SHuffle and E. coli BL21 (DE3)

#### 4.3.1. Induction with IPTG

We selected two cellular densities (OD_600_ 0.6 and 0.8) and two concentrations of IPTG (0.05 mM and 0.1 mM) (Bioline, London, UK) for the growth of *E. coli* BL21 (DE3) and *E. coli* SHuffle with the plasmid dual containing *lipA* and *lif.* Thus, cultures with 10 mL of LB broth supplemented with glucose 1% (*w*/*v*) and chloramphenicol (50 µg/mL) were inoculated with overnight cultures of both strains (1%) and incubated at 30 °C and 150 rpm. After induction, the cultures were incubated again, but at 18 °C and 100 rpm. The expression of the proteins of each culture was followed at 2, 4, and 6 h by an SDS-PAGE analysis of the aliquots of the whole culture. The cultures were harvested after 6 h and frozen at −20 °C until the measurement of their enzyme activities. Each experiment was performed in triplicate, and the results were indicated as the average of three measurements. 

#### 4.3.2. Inducing with Autoinducing Medium

We prepared an autoinducing broth according to the Studier ZYM 5052 recipe [31]. The growth curves for both strains were followed by measurements of OD_600_ every 2 h and for up to 96 h. Different percentages of inocula grown overnight (1%, 2%, 4%, 8%, and 10% *v*/*v*) at 18 °C and with moderate agitation were used (Bioscreen C BMR analyzer growth test; Thermo Electron, Hanau, Germany). The inocula cultures were grown overnight at 30 °C in a modified non-inducing broth MDG, including yeast extract (1%) and without aspartate, to avoid the early expression of the recombinant genes [32].

To monitor the enzyme expression over time for each strain, we prepared 14 cultures of 10 mL in flasks of 100 mL which were inoculated at 4% with overnight cultures in non-inducing broth. The flasks were incubated at 18 °C and 100 rpm. One flask was taken at a specific time to evaluate the optical density, SDS-PAGE profile, and lipase activity. One milliliter was taken for SDS-PAGE and frozen until use. The remaining culture was separated by centrifugation (8000 rpm, 4 °C for 15 min) and the pellets were frozen, thawed, and lysed with a lysis buffer (Tris HCl 20 mM pH 8.2, glycerol 5% *v*/*v*, NaCl 200 mM, CaCl_2_ 5 mM, lysozyme 100 µg/mL, Triton 0.3%, and phenylmethylsulfonyl fluoride (PMSF) 0.2 mM). Six milliliters of buffer was used per gram of wet cells and incubated for 30 min at 37 °C with a sporadic vortex. 

The optical density at 600 nm (Perkin Elmer UV/Vis spectrophotometer Lambda 35), activity (units of activity per mL), and specific activity (U/mg protein) were obtained for each culture. The experiment was replicated two times more for each *E. coli* strain, and the results were expressed as the average.

### 4.4. SDS-PAGE Analysis and Protein Concentration

We followed the lipase expression by 12.5% SDS-PAGE according to the Laemmli method [52]. Aliquots of the whole culture were collected at specific times before induction and after induction with IPTG; 20 μL were boiled with loading buffer 5x and loaded in each lane. The cells obtained from 1 mL of the whole culture were harvested and lysed as above in the experiments using autoinducing media. Soluble and insoluble fractions were separated by centrifugation at 14,000 rpm for 20 min. A volume equivalent to 10 μg from the soluble fraction was loaded in each lane. The pellets from insoluble fractions were homogenized with 100 μL of Tris HCl 20 mM pH 8.2 and 1% SDS and boiled for 12 min. Five microliters of this insoluble fraction homogenate was boiled again with loading buffer. All the samples were adjusted to the same volume with loading buffer and loaded. The molecular weight of lipase was estimated by comparison with the Opti-Protein XL marker G266 (ABM, Richmond, BC, Canada). The quantification of proteins was performed with the modified Bradford method at 590 nm and 450 nm [53].

### 4.5. Determination of Lipase Activity

**Figure A2:**
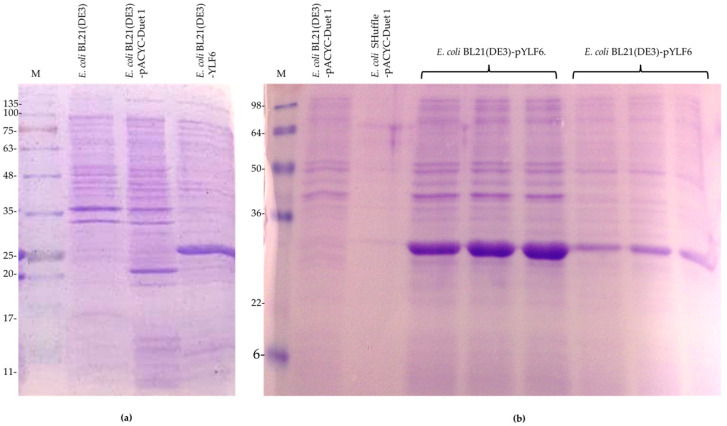
SDS PAGE protein profile of aliquots of whole cultures from *E. coli* strains after induction with IPTG 0.05 mM: (**a**) M: Opti-Protein XL G266 (ABM Inc, Richmond, BC, Canada). 1. *E. coli* BL21(DE3). 2. *E. coli* BL21 (DE3)-pACYC. 3. *E. coli* BL21 (DE3)-pYLF6 (**b**) M: Protein marker. 1. *E. coli* BL21(DE3)-pACYC. 2. *E. coli* SHuffle-pACYC. 3. 4. 5. *E. coli* BL21 (DE3)-pYLF6 clones. 6. 7. 8. *E. coli* SHuffle - pYLF6 clones.

The activity of the soluble lysates (U/mL) was found with a microplate spectrophotometer (Imark, Bio-Rad, Hercules, CA, USA) by using p-nitrophenyl palmitate 1 mM as the substrate (pNPP). We followed the methodology published by Selvin, using an absorbance of 415 nm with 10 µL of the soluble lysate in a total volume of reaction of 250 µL [54]. As a control of the lipase activity and quality of reagents, we used a dilution (1/1000) of the commercial recombinant enzyme from *Rhizomucor miehei* (Millipore, Sigma). Similarly, lysates from *E. coli* BL21 (DE3) and *E. coli* SHuffle, both transformed with pACYC Duet-1, were used as negative controls (Figure A2, Appendix A). The molar absorptivity coefficient of p-nitrophenol, using, as buffer, Tris HCl 20 mM pH 8.2, Triton 0.3% (*v*/*v*), and CaCl_2_ 5 mM, was established as 1.793 × 10^4^ M^−1^ cm^−1^. One unit of lipase was defined as the amount of enzyme able to produce 1 nmol of p-nitrophenol per minute at 37 °C and pH 8.2. 

### 4.6. Evaluation of the Stability of Recombinant LipA at Different Temperatures

The stability at different temperatures of the enzyme LipA produced for both hosts was performed with a PCR thermocycler using a temperature gradient between 40 °C and 64 °C (Bio-Rad, Hercules, CA, USA), as previously established but with some modifications [55]. Tubes of 0.2 mL with 100 µL of enzyme solution were placed in six rows, each one at a different temperature (40 °C, 45 °C, 50 °C, 55 °C, 60 °C, and 64 °C). The enzyme solutions were cooled at room temperature after one hour of incubation, and 10 µL were poured into 240 µL of reaction buffer; the activity was obtained as described. The results were expressed as the residual activity, considering the activity at 40 °C as 100%. In a parallel assay, a dilution of the commercial thermotolerant lipase from *R. miehei* was used as the control of the thermal resistance at different temperatures.

### 4.7. Evaluation of the Stability of Recombinant LipA in Methanol and Ethanol

We assessed the stability of the LipA produced by each strain in methanol and ethanol, as stated by Dror et al. with slight modifications [50]. The activity was followed for 60 min by microplates, using a final volume of 250 µL and two concentrations of methanol (50% and 60%) and ethanol (70% and 85%). A total volume of 4 mL was used for every assay, mixing 600 µL of lipase solution, buffer Tris HCl 20 mM pH 8.2, and the solvent at the concentrations above indicated. The lipase solution was incorporated last. Aliquots of 50 µL of each of the above solutions (equivalent to 10 µL of lipase) were mixed with 200 µL of the reaction buffer, and the activities were calculated from the beginning and every ten min up to 1 h. The precipitates were removed by centrifugation. The residual activity was calculated for each enzyme, with the activity of lipase without alcohol as 100%. The half-life values were calculated for the methanol curves according to the decay exponential model [50].

### 4.8. Molecular Dynamics Simulations of LipA with and without SS-Bridge

The molecular simulations were performed using the Gromacs software package version 2016.5 [56]. The analysis was performed using Gromacs analysis tools, and the trajectories were visualized using VMD (Visual Molecular Dynamics) [57]. The starting structure was taken from the Protein Data Bank (PDB) [58]. We used entry 1EX9, which corresponds to the enzyme conformation with the reaction site open [34]. The protein was solvated using the TIP3P water model [59], and the Amber force field ff99SB*-ILDN was used, which is an iteratively modified version of ff99 which is known to describe protein interactions accurately [60]. Two versions of LipA were used, one with the SS-bridge modeled as the LipA expressed from *E. coli* SHuffle T7 and another one without the SS-bridge representing the LipA expressed from *E. coli* BL21 (DE3). Simulations were performed using temperature and pressure coupling at a temperature and pressure of 300 K and 1 bar, respectively. Temperature coupling was done using the v-rescale [61]. Thermostat and pressure coupling used the Parrinello–Rahman barostat [62]. Long-range Coulomb interactions were calculated using Particle-Mesh-Ewald [63], and bonds were constrained using the Linear Constraint Solver (LINCS) [64]. Simulations were performed using periodic boundary conditions with a time step of 2 fs. We first equilibrated the system for 100 picoseconds (ps) using NVT conditions (Constant number, volume and temperature) then 100 ps of NPT equilibration (Constant number, pressure and temperature), and finally performed the production simulations for 1 µs. We also performed a 1 µs simulation of LipA without an SS-bridge, starting from a closed conformation obtained from the 1 µs simulation of LipA with an SS-bridge.

## 5. Conclusions

The cloning of the *lipA* gene without its signal peptide and a fragment of its chaperone gene in the same plasmid can be considered a valid strategy for the soluble and active heterologous expression of LipA. Furthermore, it is worth considering the characteristics of the expression host, which preserve the enzyme properties present in the native bacteria. In this study, *E. coli* SHuffle proved to be a strain able to express LipA with superior performance compared to the enzyme produced by *E. coli* BL21 (DE3). The use of the autoinduction medium represents an alternative to express the two genes, such as *lipA* and *lif*, regulated by lac promoters that do not require continuous monitoring and avoid the use of high-cost reagents such as IPTG. Furthermore, the expression system evaluated with an autoinducing medium without lactose is an option to modulate even more the expression of genes under the control of strong promoters such as T7, giving a more sustained expression of the enzyme.

## Figures and Tables

**Table 1:** Specific activity for each strain in autoinducing medium ZYM 5052, with and without lactose.

	*E. coli* SHuffle		*E. coli* BL21 (DE3)	
Time (h)	Specific Activity U/mg with Lactose	Specific Activity U/mg without Lactose	Specific Activity U/mg with Lactose	Specific Activity U/mg without Lactose
9	178 ± 0.2	112 ± 10	205 ± 21	143 ± 28
13	282 ± 1	197 ± 1	252 ± 21	338 ± 36
18	256 ± 26	202 ± 6	246 ± 20	267 ± 13
30	365 ± 20	463 ± 15	283 ± 17	354 ± 41
36	543 ± 29	465 ± 26	243 ± 5	349 ± 22

Values are means ± standard deviation from three assays.

**Table 2:** Primers used in this study.

Primer	Sequence	Feature
PafoR	5′-ATGAAGAAGAAGTCTCTGCTCC-3′	Amplification of the whole sequence of *lipA*.
Parev	5′-CTACAGGCTGGCGTTCTTCAG-3′	Amplification of the whole sequence of *lipA*.
Folfor	5′-ATGGTGCCGGCCCCCCAGGTCATG-3′	Amplification of the whole sequence of *lif*.
Perm	5′-TCAGCGCTGCTCGGCCTGGCGCAT-3′	Amplification of the whole sequence of *lif.* [17];
NdelipF	5′-GGAATTCCATATGAGCACCTACACCCAGACC-3′	Amplification of *lipA* without signal peptide; recognition site for *Nde*I.
XholipR	5′-CCGCTCGAGCTACAGGCTGGCGTTCTTCAG-3′	Amplification of *lipA* without signal peptide; recognition site for *Xho*I;
Ncofol	5′-CATGCCATGGTGCCGGCCCCCCAGGTCATG-3′	Amplification of *lif* without inner membrane anchor; recognition site for *Nco*I.
PBR	5′-CGATAAGCTTTCAGCGCTGCTCGGCCTGG-3′	Amplification of *lif;* recognition site for *Hind*III;

## Abbreviations

DMSO	Dimethyl sulfoxide
IPTG	Isopropyl -β- d-1-thiogalactopyranoside
PCR	Polymerase chain reaction
MCS	Multi cloning site
CFU	Colony-forming unit
OD_600_	Optical density at 600 nm
KDa	Kilodalton
PMSF	Phenylmethyl sulfonyl fluoride
Ps	Picoseconds
SDS-PAGE	Phenylmethyl sulfonyl fluoride
MD	Molecular dynamics
K	Kelvin
μL	Microliter
NVT	Constant number, volume and temperature
NPT	Constant number, pressure and temperature
fs	Femtoseconds

## References

[1] Hasan F., Shah A.A., Hameed A. (2006). Industrial applications of microbial lipases. Enzyme Microb. Technol..

[2] Houde A., Kademi A., Leblanc D. (2004). Lipases and Their Industrial Applications: An Overview. Appl. Biochem. Biotechnol..

[3] Andualema B., Gessesse A. (2012). Microbial lipases and their industrial applications: Review. Biotechnology.

[4] Sarrouh B., Santos T.M., Miyoshi A., Dias R., Azevedo V. (2012). Up-To-Date Insight on Industrial Enzymes Applications and Global Market. J. Bioprocess. Biotech..

[5] Jaeger K., Liebeton K., Zonta A., Schimossek K., Reetz M.T. (1996). Biotechnological application of *Pseudomonas aeruginosa* lipase: Efficient kinetic resolution of amines and alcohols. Appl. Microbiol. Biotechnol..

[6] Kanwar S.S., Verma M.L., Maheshwari C., Chauhan S., Chimni S.S., Chauhan G.S. (2006). Properties of Poly (AAc-co-HPMA-cl-EGDMA) Hydrogel-Bound Lipase of *Pseudomonas aeruginosa* MTCC-4713 and Its Use in Synthesis of Methyl Acrylate. J. Appl. Polym. Sci..

[7] Liebeton K., Zacharias A., Jaeger K.E. (2001). Disulfide bond in *Pseudomonas aeruginosa* lipase stabilizes the structure but is not required for interaction with its foldase. J. Bacteriol..

[8] Urban A., Leipelt M., Eggert T., Jaeger K.E. (2001). DsbA and DsbC affect extracellular enzyme formation in *Pseudomonas aeruginosa*. J. Bacteriol..

[9] Jaeger K., Eggert T. (2002). Lipases for biotechnology. Curr. Opin. Biotechnol..

[10] Wu X., You P., Su E., Xu J., Gao B., Wei D. (2012). In vivo functional expression of a screened *P. aeruginosa* chaperone-dependent lipase in *E. coli*. BMC Biotechnol..

[11] Madan B., Mishra P. (2010). Co-expression of the lipase and foldase of *Pseudomonas aeruginosa* to a functional lipase in *Escherichia coli*. Appl. Microbiol. Biotechnol..

[12] Ogino H., Katou Y., Akagi R., Mimitsuka T., Hiroshima S., Gemba Y., Doukyu N., Yasuda M., Ishimi K., Ishikawa H. (2007). Cloning and expression of gene, and activation of an organic solvent-stable lipase from P*seudomonas aeruginosa* LST-03. Extremophiles.

[13] Xu Y., Yasin A., Tang R., Scharer J.M., Moo-Young M., Chou C.P. (2008). Heterologous expression of lipase in *Escherichia coli* is limited by folding and disulfide bond formation. Appl. Microbiol. Biotechnol..

[14] Rosano G.L., Ceccarelli E.A. (2014). Recombinant protein expression in *Escherichia coli*: Advances and challenges. Front. Microbiol..

[15] Rosenau F., Jaeger K. (2000). Bacterial lipases from *Pseudomonas:* regulation of gene expression and mechanisms of secretion. Biochimie.

[16] Hobson A.H., Buckley C.M., Aamand J.L., Jørgensen S.T., Diderichsen B., McConnell D.J. (1993). Activation of a bacterial lipase by its chaperone. Proc. Natl. Acad. Sci. USA.

[17] Peng R., Lin J., Wei D. (2011). Co-expression of an organic solvent-tolerant lipase and its cognate foldase of *Pseudomonas aeruginosa* CS-2 and the application of the immobilized recombinant lipase. Appl. Biochem. Biotechnol..

[18] Akbari N., Khajeh K., Ghaemi N., Salemi Z. (2010). Efficient refolding of recombinant lipase from *Escherichia coli* inclusion bodies by response surface methodology. Protein Expr. Purif..

[19] Quyen D.T., Schmidt-Dannert C., Schmid R.D. (1999). High-level formation of active *Pseudomonas cepacia* lipase after heterologous expression of the encoding gene and its modified chaperone in *Escherichia coli* and rapid in vitro refolding. Appl. Environ. Microbiol..

[20] Kadokura H., Katzen F., Beckwith J. (2003). Protein Disulfide Bond Formation in Prokaryotes. Annu. Rev. Biochem..

[21] Lobstein J., Emrich C.A., Jeans C., Faulkner M., Riggs P., Berkmen M. (2012). SHuffle, a novel *Escherichia coli* protein expression strain capable of correctly folding disulfide bonded proteins in its cytoplasm. Microb. Cell Fact..

[22] Ren G., Ke N., Berkmen M. (2016). Use of the SHuffle Strains in Production of Proteins. Current Protocols in Protein Science.

[23] Ogino H., Ishikawa H. (2001). Enzymes which are stable in the presence of organic solvents. J. Biosci. Bioeng..

[24] Ogino H., Uchiho T., Yokoo J., Kobayashi R., Ichise R., Ishikawa H. (2001). Role of intermolecular disulfide bonds of the organic solvent-stable PST-01 protease in its organic solvent stability. Appl. Environ. Microbiol..

[25] Kumar A., Dhar K., Kanwar S.S., Arora P.K. (2016). Lipase catalysis in organic solvents: Advantages and applications. Biol. Proced. Online.

[26] Han Z.L., Han S.Y., Zheng S.P., Lin Y. (2009). Enhancing thermostability of a *Rhizomucor miehei* lipase by engineering a disulfide bond and displaying on the yeast cell surface. Appl. Microbiol. Biotechnol..

[27] Yu X.W., Tan N.J., Xiao R., Xu Y. (2012). Engineering a Disulfide Bond in the Lid Hinge Region of *Rhizopus chinensis* Lipase: Increased Thermostability and Altered Acyl Chain Length Specificity. PLoS One.

[28] Le Q.A.T., Joo J.C., Yoo Y.J., Kim Y.H. (2012). Development of thermostable *Candida antarctica* lipase B through novel in silico design of disulfide bridge. Biotechnol. Bioeng..

[29] Godoy C.A., Klett J., Di Geronimo B., Hermoso J.A., Guisán J.M., Carrasco-López C. (2019). Disulfide engineered lipase to enhance the catalytic activity: A structure-based approach on btl2. Int. J. Mol. Sci..

[30] Sørensen H.P., Mortensen K.K. (2005). Advanced genetic strategies for recombinant protein expression in *Escherichia coli*. J. Biotechnol..

[31] Terpe K. (2006). Overview of bacterial expression system for heterologous protein production: From molecular and biochemical fundamentals to commercial systems. Appl. Microbiol. Biotechnol..

[32] Studier F.W. (2005). Protein production by auto-induction in high-density shaking cultures. Protein Expr. Purif..

[33] Zhang Z., Kuipers G., Niemiec Ł., Baumgarten T., Slotboom D.J., De Gier J., Hjelm A. (2015). High-level production of membrane proteins in *E. coli* BL21(DE3) by omitting the inducer IPTG. Microb. Cell Fact..

[34] Ogino H., Miyamoto K., Yasuda M., Ishimi K., Ishikawa H. (1999). Growth of organic solvent-tolerant *Pseudomonas aeruginosa* LST-03 in the presence of various organic solvents and production of lipolytic enzyme in the presence of cyclohexane. Biochem. Eng. J..

[35] Nardini M., Lang D.A., Liebeton K., Jaeger K.E., Dijkstra B.W. (2000). Crystal Structure of *Pseudomonas aeruginosa* Lipase in the open conformation. The Prototype for Family I.1 of Bacterial Lipases. J. Biol. Chem..

[36] Thiruvengadam K., Baskaran S.K., Pennathur G. (2018). Understanding domain movements and interactions of *Pseudomonas aeruginosa* lipase with lipid molecule tristearoyl glycerol: A molecular dynamics approach. J. Mol. Graph. Model..

[37] Kawata T., Ogino H. (2010). Amino acid residues involved in organic solvent-stability of the LST-03 lipase. Biochem. Biophys. Res. Commun..

[38] Liebeton K., Zonta A., Schimossek K., Nardini M., Lang D., Dijkstra B.W., Reetz M.T., Jaeger K.E. (2000). Directed evolution of an enantioselective lipase. Chem. Biol..

[39] Smith R.S., Iglewski B.H.P. (2003). *P. aeruginosa* quorum-sensing systems and virulence. Curr. Opin. Microbiol..

[40] Ogino H., Inoue S., Yasuda M., Doukyu N. (2013). Hyper-activation of foldase-dependent lipase with lipase-specific foldase. J. Biotechnol..

[41] Quyen T.D., Vu C.H., Thu Le G.T. (2012). Enhancing functional production of a chaperone-dependent lipase in *Escherichia coli* using the dual expression cassette plasmid. Microb. Cell Fact..

[42] West S.E., Iglewski B.H. (1988). Codon usage in *Pseudomonas aeruginosa*. Nucleic Acids Res..

[43] Briand L., Marcion G., Kriznik A., Heydel J.M., Artur Y., Garrido C., Seigneuric R., Neiers F. (2016). A self-inducible heterologous protein expression system in *Escherichia coli*. Sci. Rep..

[44] Nair R., Salvi P., Banerjee S., Raiker V.A., Bandyopadhyay S., Soorapaneni S., Kotwal P., Padmanabhan S. (2009). Yeast extract mediated autoinduction of lacUV5 promoter: An insight. N. Biotechnol..

[45] Mercatelli E., Barbieri L., Luchinat E., Banci L. (2016). Direct structural evidence of protein redox regulation obtained by in-cell NMR. Biochim. Biophys. Acta - Mol. Cell Res..

[46] de Marco A. (2009). Strategies for successful recombinant expression of disulfide bond-dependent proteins in *Escherichia coli*. Microb. Cell Fact..

[47] Calce E., Vitale R.M., Scaloni A., Amodeo P., De Luca S. (2015). Air oxidation method employed for the disulfide bond formation of natural and synthetic peptides. Amino Acids.

[48] Haworth N.L., Gready J.E., George R.A., Wouters M.A. (2007). Evaluating the stability of disulfide bridges in proteins: A torsional potential energy surface for diethyl disulfide. Mol. Simul..

[49] Zhang Y., Schulten K., Gruebele M., Bansal P.S., Wilson D., Daly N.L. (2016). Disulfide bridges: Bringing together frustrated structure in a bioactive peptide. Biophys. J..

[50] Dror A., Shemesh E., Dayan N., Fishman A. (2014). Protein Engineering by Random Mutagenesis and Structure-Guided Consensus of *Geobacillus stearothermophilus* Lipase T6 for Enhanced Stability in Methanol. Appl. Environ. Microbiol..

[51] Sambrook J., Russell D.W. (2012). Molecular Cloning A Laboratory Manual.

[52] Laemmli U.K. (1970). Cleavage of structural proteins during the assembly of the head of bacteriophage T4. Nature.

[53] Ernst O., Zor T. (2010). Linearization of the Bradford Protein Assay. J. Vis. Exp..

[54] Selvin J., Kennedy J., Lejon D.P.H., Kiran G.S., Dobson A.D.W. (2012). Isolation identification and biochemical characterization of a novel halo-tolerant lipase from the metagenome of the marine sponge *Haliclona simulans*. Microb. Cell Fact..

[55] Xie Y., An J., Yang G., Wu G., Zhang Y., Cui L., Feng Y. (2014). Enhanced Enzyme Kinetic Stability by Increasing Rigidity within the Active Site. J. Biol. Chem..

[56] Van Der Spoel D., Lindahl E., Hess B., Groenhof G., Mark A.E., Berendsen H. (2005). GROMACS: Fast, Flexible, and Free. J. Comput. Chem..

[57] Humphrey W., Dalke A., Schulten K. (1996). VMD: Visual Molecular Dynamics. J. Mol. Graph..

[58] Burley S.K., Berman H.M., Kleywegt G.J., Markley J.L., Nakamura H., Velankar S. (2017). Protein Data Bank (PDB): The Single Global Macromolecular Structure Archive. Methods Mol. Biol..

[59] Jorgensen W.L., Chandrasekhar J., Madura J.D., Impey R.W., Klein M.L. (1983). Comparison of simple potential functions for simulating liquid water. J. Chem. Phys..

[60] Hornak V., Abel R., Okur A., Strockbine B., Roitberg A., Simmerling C. (2006). Comparison of multiple AMBER force fields and development of improved protein backbone parameters. Proteins.

[61] Bussi G., Donadio D., Parrinello M. (2007). Canonical sampling through velocity rescaling. J. Chem. Phys..

[62] Parrinello M., Rahman A. (1981). Polymorphic transitions in single crystals: A new molecular dynamics method. J. Appl. Phys..

[63] Essmann U., Perera L., Berkowitz M.L., Darden T., Lee H., Pedersen L.G. (1995). A smooth particle mesh Ewald method. J. Chem. Phys..

[64] Hess B., Bekker H., Berendsen H.J.C., Fraaije J.G.E.M. (1997). LINCS: A Linear Constraint Solver for Molecular Simulations. J. Comput. Chem..

